# Proposal of a therapeutic algorithm for the psychopharmacological management of treatment-resistant depression

**DOI:** 10.1192/j.eurpsy.2022.1451

**Published:** 2022-09-01

**Authors:** A. Sanz Giancola, M.D.C. Molina Liétor, I. Cuevas Iñiguez, C. Alvarez Garcia, M. Blanco Prieto

**Affiliations:** Hospital Universitario Príncipe de Asturias, Psychiatry, Alcalá de Henares, Spain

**Keywords:** TRD, psychopharmacological, evidence-based, algorithm

## Abstract

**Introduction:**

The lack of a standardised definition for the concept of TRD and an adequate criteria for therapeutic response make difficult the management of patients with MDD who do not achieve remission with one or more courses of treatment. All classifications suggested to define TRD are arbitrary, partially evidence-based, subordinated to the pharmacological findings of the time in which they are written and with serious inconsistencies, making it difficult to construct a universal and enduring diagnostic system.

**Objectives:**

Considering that the most important goal in treating a patient with Major Depressive Disorder (MDD) should be remission and return to previous functionality, the search for a standardised, evidence-based classification system will allow timely and effective interventions leading to the reduction of this devastating condition.

**Methods:**

Bibliographic review

**Results:**

The proposed therapeutic algorithm arises from the combination of several fundamental principles for the management of treatment-resistant depression: the different classification systems of the concept, as well as the concepts of response, relapse, recurrence and remission; the scientific evidence found in the current literature, routine clinical practice, knowledge of switching and augmentation strategies, the new pharmacological targets and neurobiological hypothesis discovered, without forgetting finally the different clinical profiles of depressive symptomatology and the specific indications of each antidepressant.

**Conclusions:**

Resistant depression is difficult to treat successfully and is not a uniform entity. Recently there has been a move to characterise treatment-resistant depression as ‘difficult-to-treat’ depression on the basis that the former description implies that depression treatments are normally effective and that non-response is therefore somehow abnormal.

**Disclosure:**

No significant relationships.

